# Regulatory Mechanisms and Safety Evaluation of Exogenous Progesterone for Suppression of Rutting Behavior in Male Sika Deer (*Cervus nippon*)

**DOI:** 10.3390/ani16030488

**Published:** 2026-02-04

**Authors:** Peize Du, Xinyu Peng, Huansheng Han, Fanzhi Kong, Lieping Zhao, Zhen Zhang, Liying Sun, Wenxi Qian

**Affiliations:** 1 College of Animal Science and Veterinary Medicine, Heilongjiang Bayi Agricultural University, Daqing 163319, China; 2 Heilongjiang Province Agricultural Reclamation Academy, Harbin 150431, China; 3 Genhe Forestry Industry Co., Ltd., Inner Mongolia Forest Industry Group, Genhe 021000, China; 4 School of Animal Science and technology, Tarim University, Alar 843300, China

**Keywords:** *Cervus nippon*, sika deer, exogenous progesterone, rutting behavior inhibition, hormonal regulation, untargeted metabolomics

## Abstract

Managing rutting aggression is critical in sika deer (*Cervus nippon*) farming. To mitigate rutting aggression in male sika deer, this study evaluated the efficacy, safety, and physiological mechanisms of exogenous progesterone. Twelve sika deer were randomly assigned to either a control group or a treatment group, with behavior monitored for 60 days post-administration. Serum hormones, non-targeted serum metabolomics, biochemical indicators (including reflecting liver and kidney function), and subsequent antler performance were assessed. The treatment group exhibited significantly reduced aggressive and mating behavior throughout the study (*p* < 0.05). HPG axis hormones (GnRH, LH, FSH, and T) and PRL were significantly reduced throughout the study (*p* < 0.05), while TRH was elevated, T4 declined, and GH showed time-dependent fluctuations. Differential metabolites were significantly enriched in pathways related to nucleotide metabolism, pyruvate metabolism, and arachidonic acid metabolism. Except for a transient decrease in the ALB/GLB ratio (*p* < 0.05), no significant changes were observed in other biochemical indicators or antler performance (*p* > 0.05). This study confirms that exogenous progesterone effectively controls rutting behavior primarily via HPG-axis suppression and multi-system endocrine interactions, without inducing detectable organ toxicity or compromising production, supporting its use as a safe management intervention.

## Simple Summary

Aggression among adult male sika deer during the rutting season causes injuries, fatalities, and economic losses in high-density farming, posing significant animal welfare challenges. We assessed whether a single injection of long-acting progesterone could safely and effectively suppress these behaviors. Twelve deer were randomly assigned to a treatment group (one 330 mg subcutaneous progesterone injection) or a control group. Over a 60-day period, we monitored behavior, serum hormones and metabolites, health markers, and later antler performance. The treatment significantly reduced aggression and mating activity, linked to suppressed reproductive hormones and altered metabolism. Importantly, it had no adverse effects on organ health or antler yield. This study presents a practical hormonal strategy to improve welfare and sustainability in deer production by mitigating rutting-related aggression.

## 1. Introduction

The sika deer (*Cervus nippon*) is an important economic species and plays a key role in the global deer antler industry. The global market for deer antler extracts was valued at USD 192 million in 2024 and is projected to reach USD 289 million by 2033 [1]. However, the rutting season presents major challenges in sika deer production systems. During this period, heightened aggressiveness and territorial behavior among males frequently result in group fighting, injuries, and occasional mortality. This reduces antler yield and imposes substantial economic burdens on farmers through increased medical expenses, animal replacement costs, and risks to personnel safety [2].

Sika deer farming is primarily concentrated in Asia, with China serving as the leading producer. Sika deer supply high-value velvet antler for traditional medicine and health products, support advanced food processing, and contribute to an extended supply chain [3]. The rutting period is a critical phase in the management of male sika deer and is regulated by the hypothalamic–pituitary–gonadal (HPG) axis. During this period, luteinizing hormone (LH) levels rise significantly, stimulating testosterone (T) synthesis, which directly drives behavioral changes such as increased aggressiveness and heightened mating motivation. Concurrently, follicle-stimulating hormone (FSH) levels increase, supporting spermatogenesis and overall reproductive function [4]. Although these endocrine changes are beneficial for reproduction, they are often associated with elevated stress, increased energy expenditure, and significant body weight loss under high-density farming conditions, ultimately reducing antler yield and production efficiency [2].

Hormonal intervention has recently emerged as an effective strategy for regulating reproductive behavior in animals. Progesterone (P), a natural steroid hormone, is primarily used in females to suppress estrus and synchronize ovulation and has been widely applied in ruminants such as cattle and sheep [5]. Progesterone inhibits gonadotropin-releasing hormone (GnRH) and LH secretion via negative feedback, thereby reducing downstream hormone levels [6]. Increasing evidence suggests that progesterone also exhibits anti-androgenic effects in males. For example, exogenous progesterone has been shown to reduce aggressive behavior and regulate metabolic pathways in rodents [7]. This regulatory mechanism finds a robust physiological basis in male ruminants. In rams (*Ovis aries*), exogenous progesterone administration has been demonstrated to effectively suppress pulsatile luteinizing hormone secretion, leading to a marked decline in circulating LH concentrations [8]. Similarly, studies in bulls (*Bos taurus*) indicate that progesterone analogs can significantly reduce LH pulse frequency, with higher doses further suppressing testosterone production [9]. Notably, the pivotal role of androgens in mediating male rutting behavior has been firmly established in cervids. For instance, experiments involving red deer (*Cervus elaphus*) have shown that castration-induced testosterone deficiency eliminates rut-associated aggression, a behavioral pattern that can be fully restored through exogenous testosterone replacement [10]. Therefore, building upon the conserved endocrine pathway whereby progesterone inhibits gonadotropin secretion and downstream testosterone synthesis, we hypothesize that targeted intervention within this axis represents a physiologically grounded strategy for mitigating rut-related aggression in male cervids. In parallel, non-targeted metabolomics, a high-throughput technique for evaluating systemic metabolic responses to hormonal interventions, has been used in previous studies demonstrating changes in lipid and amino acid metabolism following exogenous estrogen treatment in mice [11].

Previous studies in cervid species have focused mainly on hormonal roles in seasonal reproduction and antler growth. For example, in male red deer, T levels peak during the rutting period (September–October), correlating with aggressiveness and mating behavior, but remain low during antler growth (spring) to facilitate energy allocation to skeletal development [12]. Similarly, in male white-tailed deer (*Odocoileus virginianus*), prolactin (PRL) exhibits a seasonal pattern, with elevated summer levels associated with antler growth and a potential inhibitory effect on reproduction by suppressing GnRH. In contrast, thyroxine (T4) contributes to energy metabolism and behavioral adaptation, with no seasonal variation [13]. Although these studies provide valuable descriptive insights into hormonal fluctuations, they do not include systematic evaluations of hormonal interventions, such as progesterone use in males. As farming intensifies, there is an urgent need to systematically evaluate the safety of exogenous progesterone in male cervids (including liver and kidney function and inflammatory responses), its effects on the reproductive endocrine axis, and potential impacts on antler performance to improve animal welfare and farming efficiency [14].

Despite the widespread use of progesterone in female ruminants for estrus suppression and ovulation synchronization, its physiological roles and regulatory mechanisms in males, particularly in cervids, remain poorly characterized. Current management of the rutting period in male sika deer primarily relies on physical methods, such as single-pen housing or elevated enclosures, to reduce fighting and injuries. However, these methods can increase stress, restrict movement, and do not address the underlying hormone-driven basis of aggressive behavior [15]. Existing research focused mainly on short-term estrus suppression in females and has rarely incorporated systematic analyses of hormonal dynamics, multi-axis endocrine monitoring (including the HPG and thyroid axes), or metabolic homeostasis in males [16]. Furthermore, the potential consequences of progesterone intervention on subsequent antler yield in cervids have not been evaluated, which limits its practical adoption in farming systems.

In this study, we investigated the inhibitory effects of exogenous progesterone on rutting behavior in male sika deer and explored the underlying regulatory mechanisms. Specifically, we examined associations among behavioral changes, circulating multi-hormone profiles, and non-targeted metabolomic signatures, while simultaneously assessing biochemical safety indicators and antler performance. We hypothesize that exogenous progesterone suppresses the HPG axis through negative feedback, reducing key reproductive hormones (e.g., LH, FSH, and T). As a result, rutting-related behaviors are alleviated, adaptive metabolic adjustments are induced, and no significant adverse effects on organ function or antler production occur.

To test this hypothesis, the study utilized the following objectives: (1) to evaluate changes in aggressive and mating behavior scores and determine the regulatory effects of exogenous progesterone; (2) to measure dynamic changes in multi-axis hormones including, GnRH, LH, FSH, PRL, T, P, E2, TRH, T4, DA, and GH, and to analyze their associations with behavioral outcomes; (3) to identify differential metabolites and enriched pathways using non-targeted metabolomics to link metabolic and hormonal responses; (4) to assess treatment safety via biochemical indicators and effects on antler performance.

This study provides a safe and effective method for regulating rutting behavior in male sika deer, with potential global applications in farming.

## 2. Materials and Methods

### 2.1. Experimental Design

**Figure 1:**
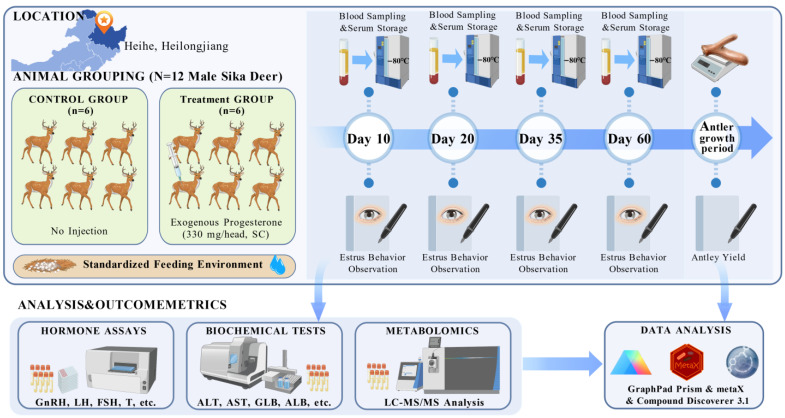
Schematic diagram of the experimental design. The control group received no injection, while the treatment group received a single subcutaneous injection of exogenous progesterone at 330 mg per stag at the onset of the rutting period. Arrows indicate the time points for behavioral observation and blood sampling at 10, 20, 35, and 60 days post-injection. Biochemical indicators were measured only at 35 and 60 days. Antler performance was tracked following the injection. Created with BioGDP.com [19].

This study was conducted at a sika deer farm in Heihe City, Heilongjiang Province, China Animals were maintained under standardized, sex-segregated captive breeding conditions. Each pen had a total area of 210 m^2^, consisting of a 90 m^2^ sheltered area and a 120 m^2^ outdoor exercise yard. During the rutting period in September 2023, twelve clinically healthy male sika deer aged five years were selected. All animals had comparable body weights (122.29 ± 8.96 kg) and a rutting behavior score of 2, as defined by established scoring criteria. Using a completely randomized design, the deer were randomly assigned to two experimental groups (*n* = 6 per group): a control group that received no injection and a treatment group that received a single subcutaneous injection of exogenous progesterone at 330 mg per animal. Blood samples were collected from the jugular vein of all animals in the morning under fasting conditions at 10, 20, 35, and 60 days post-injection administration. Serum was obtained by centrifugation and stored at −80 °C until subsequent analyses. Rutting behaviors were observed and scored at 10, 20, 35, and 60 days post-treatment. To evaluate potential effects of progesterone on antler performance, antler casting dates, harvesting dates, and fresh velvet antler weights were recorded during the 2024 production season. Throughout the experiment, both groups were housed in adjacent pens under identical environmental conditions and management protocols, with ad libitum access to water and feed provided twice daily (morning and evening) (Figure 1). All procedures were performed in strict accordance with animal welfare guidelines, complying with the National Standard of the People’s Republic of China “Laboratory Animal—Guideline for Ethical Review of Animal Welfare” (GB/T 35892-2018) and the standard “Deer Welfare” (T/CAAA 041-2020) [17,18].

### 2.2. Rutting Behavior Evaluation

Before the experiment, rutting behaviors of sika deer were observed, and a scoring standard for these behaviors was developed (Table 1).

### 2.3. Hormone Assays

Serum levels of GnRH, LH, FSH, PRL, T, P, E2, TRH, T4, DA, and GH were measured using commercial double-antibody sandwich enzyme-linked immunosorbent assay (ELISA) kits (Shanghai Youxuan Biotechnology Co., Ltd., Shanghai, China). Following the manufacturer’s instructions, standards and samples were incubated with horseradish peroxidase (HRP)-conjugated detection antibodies in pre-coated wells at 37 °C for 60 min. After washing, color was developed with tetramethylbenzidine (TMB) substrate. Absorbance was measured at 450 nm on a microplate reader (Thermo Fisher, Waltham, MA, USA). Hormone concentrations were calculated from standard curves, with all curves exhibiting a coefficient of determination (R^2^) of ≥0.99. All samples were run in triplicate, and both intra- and inter-assay coefficients of variation were below 15%.

### 2.4. Non-Targeted Metabolomics Analysis

Serum samples (100 μL) were mixed with 400 μL of pre-chilled methanol and vortexed thoroughly. The mixture was incubated on ice for 5 min and centrifuged at 21,000× *g* and 4 °C for 5 min. An aliquot of the resulting supernatant was diluted with LC–MS-grade water to achieve a final methanol concentration of 53%. The diluted samples were transferred to new tubes and centrifuged at 15,000× *g* and 4 °C for 10 min. The final supernatant was then used for UHPLC–MS/MS analysis.

Chromatographic separation was performed using a Vanquish UHPLC system coupled to an Orbitrap Q Exactive™ HF mass spectrometer (Thermo Fisher, Dreieich, Germany). Metabolites were separated on a Hypersil Gold column (100 mm × 2.1 mm, 1.9 μm) with a flow rate of 0.2 mL/min over a 17 min linear gradient. For positive ion mode, mobile phase A consisted of 0.1% formic acid in water and mobile phase B was methanol; for negative ion mode, mobile phase A was 5 mM ammonium acetate (pH 9.0), and mobile phase B was methanol. The gradient program was as follows: 2% B for 1.5 min; 2–100% B over 12 min; 100% B at 14 min; 100–2% B at 14.1 min; and 2% B maintained until 17 min. The Q Exactive™ HF operated in positive/negative polarity mode with a spray voltage of 3.2 kV, a capillary temperature of 320 °C, a sheath gas flow of 40 arb, and an auxiliary gas flow of 10 arb.

### 2.5. Biochemical Indicator Assays

Biochemical indicators primarily reflect mid- to long-term changes in organ function and metabolic homeostasis, with limited early changes expected (10 and 20 days). To facilitate integration with metabolomics data, biochemical parameters were assessed at 35 and 60 days following progesterone administration. Analyses were performed on a Hitachi 7600-020 automatic biochemical analyzer (Hitachi, Ltd., Tokyo, Japan). The evaluated indicators included: (1) liver function indices, comprising ALT, AST, AST/ALT ratio, GGT, ALP, total protein (TP), albumin (ALB), globulin (GLB), ALB/GLB ratio, total bilirubin (TBIL), direct bilirubin (DBIL), and indirect bilirubin (IBIL); (2) myocardial injury markers, including CK, LDH, and α-HBDH; (3) kidney function parameters, including BUN, creatinine (CR), uric acid (UA), and CO_2_ combining power (CO_2_-CP); (4) blood lipids profiles, including TG, total cholesterol (TC), HDL cholesterol, and LDL cholesterol; (5) electrolytes and trace elements, including K, Na, Cl, Ca, Mg and P (measured via the instrument’s ISE module or enzymatic methods); Zn, and Fe (determined using compatible colorimetric kits); (6) blood glucose (GLU). All procedures strictly adhered to the manufacturer’s protocols. The instrument was calibrated using standard quality controls before sample analysis.

### 2.6. Antler Performance Evaluation

Velvet antler harvesting was conducted according to the “two-branch” standard, defined by the presence of the main beam and brow tine before the development of the second branch, in accordance with the Chinese national standard GB/T 40943-2021 (“Grading and Quality of Sika Deer Velvet Antler”) [20]. When antlers reached this standard, fresh velvet antlers were sawn at a position 1.5–2.0 cm above the pedicle. Fresh velvet antler weights were measured on an electronic balance (accuracy 0.01 g) and recorded individually. Antler yield was expressed as the mean fresh weight (kg/deer) per group to evaluate the effects of progesterone treatment. All antlers were harvested and weighed on the day they met the two-branch standard to minimize moisture loss and maturity-related variability.

### 2.7. Data Analysis

Statistical analyses were performed using GraphPad Prism 8.0. Normality of all data was first verified with the Shapiro–Wilk test (retaining data with *p* > 0.1). Hormone levels and biochemical indicators were evaluated by two–way ANOVA followed by Bonferroni post hoc tests, whereas antler production data were compared using independent-samples *t*-tests. Data are presented as mean ± standard deviation (SD), and a value of *p* < 0.05 was considered statistically significant. Metabolomics raw data were preprocessed in Compound Discoverer 3.1 and imported into metaX for PCA, PLS-DA, and OPLS-DA. Univariate *t*-tests were applied to calculate *p* values, and variable importance in projection (VIP) scores were obtained from OPLS-DA models. Metabolites with VIP ≥ 1, *p* ≤ 0.05, and |log_2_FC| ≥ 1 were defined as differential metabolites. These metabolites were annotated using databases such as KEGG (released 25 December 2024), HMDB (released 21 January 2025), and LIPID Maps (released 17 November 2021), followed by pathway enrichment analysis; pathways with *p* < 0.05 were considered significantly enriched.

## 3. Results

### 3.1. Effects of Exogenous Progesterone on Rutting Behavior in Male Sika Deer

**Figure 2:**
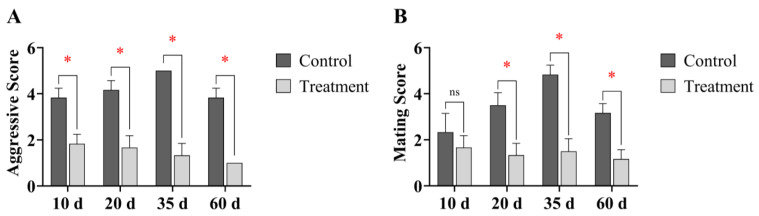
Comparison of rutting behavior scores between control and progesterone-treated male sika deer at different time points. (**A**) Aggressive scores. (**B**) Mating scores. * *p* < 0.05 compared with the control group at the corresponding time point; ns, not significant (*p* > 0.05). This notation applies to all subsequent figures.

Compared with the control group, deer in the progesterone-treated group exhibited significantly lower aggressive behavior scores at 10, 20, 35, and 60 days post-progesterone administration (*p* < 0.05, Figure 2A). Additionally, mating behavior scores were significantly reduced at 20, 35, and 60 days post-treatment (*p* < 0.05, Figure 2B). These results indicate that exogenous progesterone administration during the rutting period can effectively reduce rutting behavior in male sika deer.

### 3.2. Effects of Exogenous Progesterone on Serum Hormone Levels in Male Sika Deer

**Figure 3:**
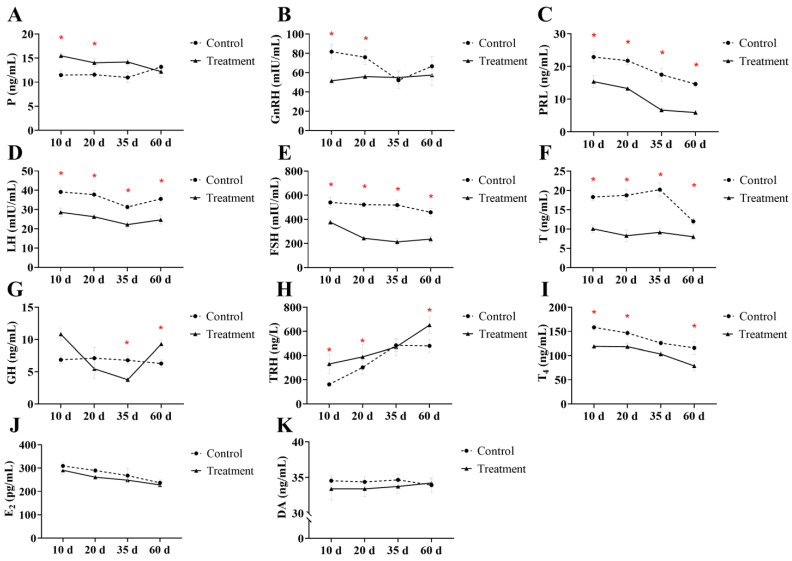
Comparison of hormone levels between the two groups of male sika deer at different time points. (**A**) Progesterone (P); (**B**) Gonadotropin-releasing hormone (GnRH); (**C**) Prolactin (PRL); (**D**) Luteinizing hormone (LH); (**E**) Follicle-stimulating hormone (FSH); (**F**) Testosterone (T); (**G**) Growth hormone (GH); (**H**) Thyrotropin-releasing hormone (TRH); (**I**) Thyroxine (T4); (**J**) Estradiol (E2); (**K**) Dopamine (DA). * indicates a significant difference between groups at the same time point (*p* < 0.05).

Hormone level changes following exogenous progesterone administration during the rutting period are presented in Figure 3. Serum GnRH and P concentrations in the treatment group differed significantly from those in the control group only at 10 and 20 days post-administration (*p* < 0.05; Figure 2A,B), with a reduction in GnRH and a corresponding increase in circulating progesterone levels. In contrast, serum PRL, LH, FSH, and T concentrations decreased significantly at all time points throughout the study (*p* < 0.05; Figure 3C–F). Growth hormone (GH) concentrations increased significantly at 35 days but decreased at 60 days (*p* < 0.05; Figure 3G). Thyrotropin-releasing hormone (TRH) and T4 concentrations showed significant differences between groups at 10, 20, and 60 days (*p* < 0.05; Figure 3H,I), with TRH elevated and T4 reduced. Dopamine (DA) and estradiol (E2) showed no significant differences at any time point throughout the study (*p* > 0.05; Figure 3J,K). These changes indicate that exogenous progesterone administration during the rutting period significantly altered serum levels of GnRH, P, PRL, LH, FSH, T, GH, TRH, and T4 in male sika deer.

### 3.3. Effects of Exogenous Progesterone on Serum Metabolome in Male Sika Deer

#### 3.3.1. Classification of Metabolites in Male Sika Deer

**Figure 4:**
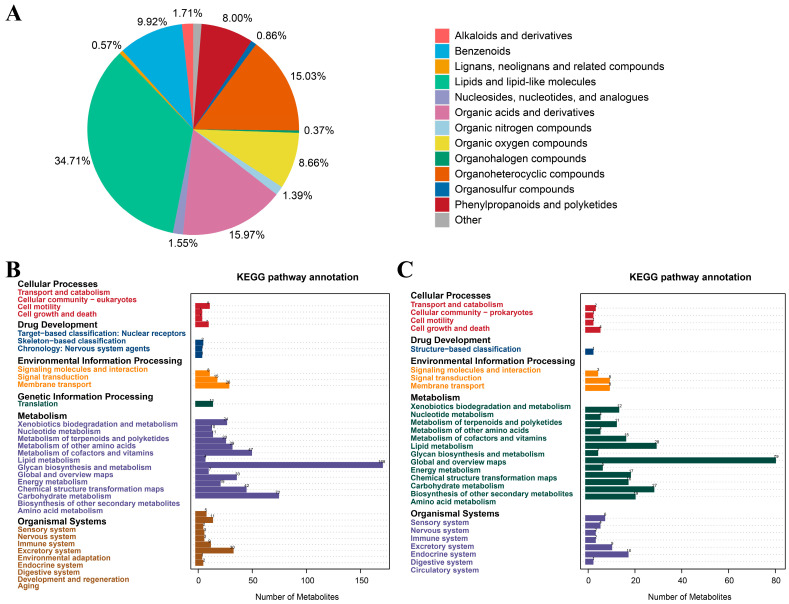
Identification and classification of all metabolites in serum samples from male sika deer. (**A**) Pie chart showing the proportional distribution of metabolite chemical classes, (**B**) KEGG functional annotation analysis of all metabolites in positive ion mode (left: enriched pathways; right: number of metabolites per pathway), (**C**) KEGG functional annotation analysis of all metabolites in negative ion mode.

Non-targeted serum metabolomic analysis identified 2449 metabolite features across both experimental groups, comprising 1492 features detected in positive ion mode and 957 in negative ion mode. The metabolome comprised lipids and lipid-like molecules (34.71%), organic acids and derivatives (15.97%), organoheterocyclic compounds (15.03%), benzenoids (9.92%), organic oxygen compounds (8.66%), phenylpropanoids and polyketides (8.00%), alkaloids and derivatives (1.71%), nucleosides, nucleotides, and analogues (1.55%), organic nitrogen compounds (1.39%), organosulfur compounds (0.86%), lignans and related compounds (0.57%), organohalogen compounds (0.37%), and others (1.26%) (Figure 4).

#### 3.3.2. Screening and Analysis of Differential Metabolites

**Figure 5:**
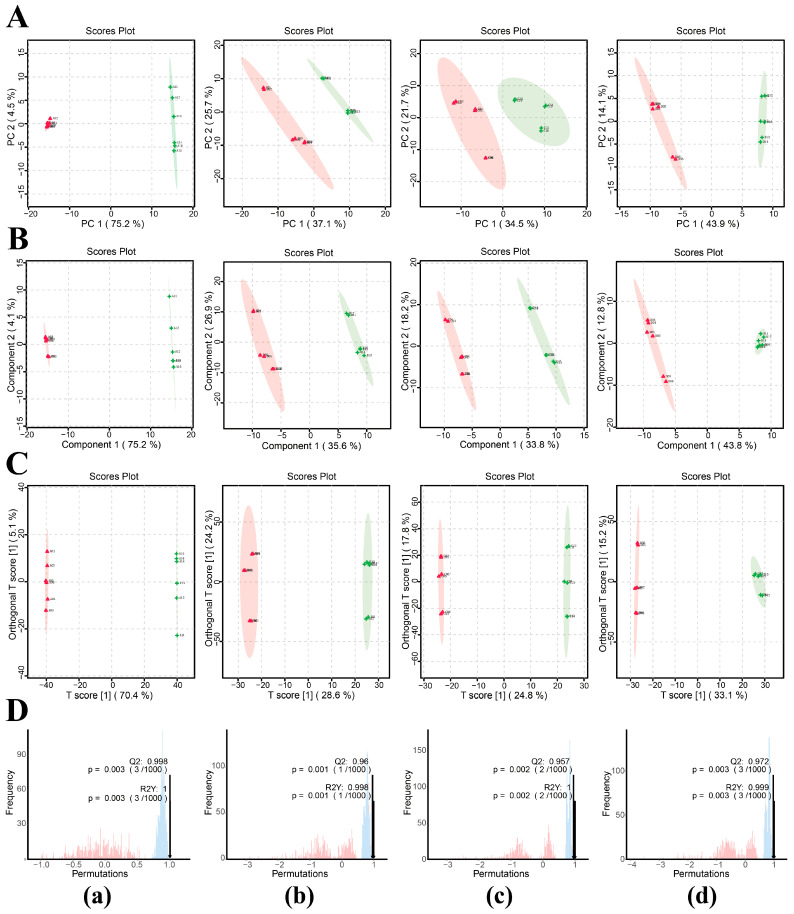
Screening of differential metabolites in serum samples from male sika deer. (**A**) PCA, (**B**) PLS-DA, and (**C**) OPLS-DA score plot (control, red; progesterone-treated, green). (**D**) OPLS-DA permutation test plot (R^2^Y, blue; Q^2^, red). R^2^Y represents model interpretation rate; Q^2^Y assesses predictive ability of the OPLS-DA model; model fits well when R^2^Y > Q^2^Y. (**a**) Comparison at 10 d, (**b**) at 20 d, (**c**) at 35 d, (**d**) at 60 d.

Principal component analysis (PCA) showed clear separation between serum samples from the control and progesterone-treated groups at all sampling time points, indicating that exogenous progesterone administration significantly altered the serum metabolic profile in male sika deer during the rutting period (Figure 5A). Partial least squares discriminant analysis (PLS-DA) further confirmed the separation between groups (Figure 5B). To eliminate noise unrelated to classification and identify metabolites responsible for significant differences, orthogonal partial least squares discriminant analysis (OPLS-DA) was employed. Predictive parameters R^2^Y and Q^2^Y under this model both exceeded 0.9, with R^2^Y exceeding Q^2^Y, demonstrating good model reliability (Figure 5C,D).

**Figure 6:**
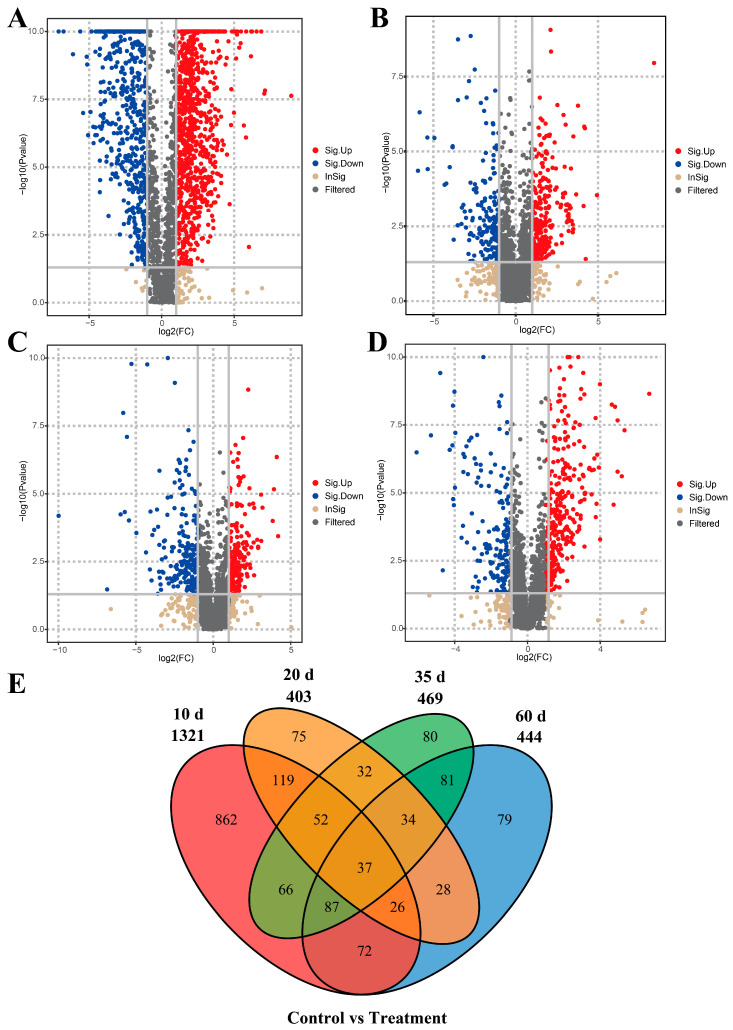
Volcano plots and Venn diagramsof differential metabolites between the two groups of male sika deer at different time points. (**A**–**D**): Volcano plots. Gray areas represent no differential expression, red areas indicate up-regulated endogenous metabolites, and blue areas indicate down-regulated endogenous metabolites. (**A**) Comparison at 10 d, (**B**) at 20 d, (**C**) at 35 d, (**D**) at 60 d; (**E**): Venn diagrams.

Based on predefined screening criteria, a total of 1730 differential metabolites were identified between the control and treatment groups across all time points (see Table 2 for details; Figure 6). Venn diagram analysis revealed 37 common differential metabolites shared across 10, 20, 35, and 60 days (Table 3). Among these, ten metabolites were involved in KEGG pathway enrichment, including Combretastatin A4, 3′-O-Methylbatatasin III, trans-Chalcone, Cortisone acetate, Andrographolide, Tricarballylic acid, 5-Fluorouridine, (ent-6α,7α)-6,7-Dihydroxy-16-kauren-19-oic acid, Lusitanicoside, and spironolactone.

#### 3.3.3. Effects of Exogenous Progesterone on Metabolic Pathways in Male Sika Deer

**Figure 7:**
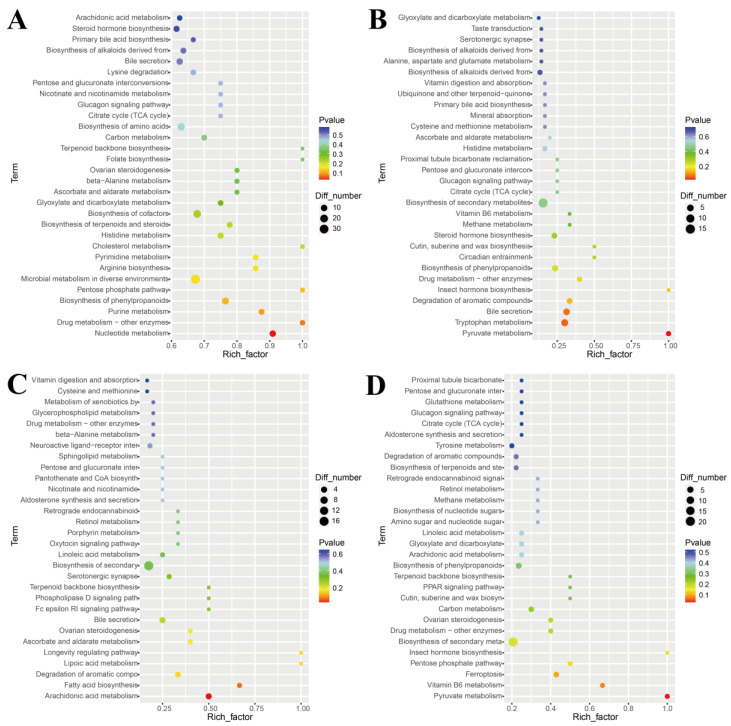
KEGG pathway analysis of differential metabolites in serum samples from male sika deer at different time points. (**A**) Top 30 KEGG-enriched pathways at 10 d; (**B**) at 20 d; (**C**) at 35 d; (**D**) at 60 d. The horizontal axis represents the enrichment factor (ratio of the proportion of differential metabolites enriched in a pathway to the background proportion); the vertical axis shows the names of enriched KEGG pathways; larger circles indicate a higher number of differential metabolites enriched in the pathway. Colors ranging from blue to red indicate *p* values from high to low (i.e., enrichment significance from low to high).

**Figure 8:**
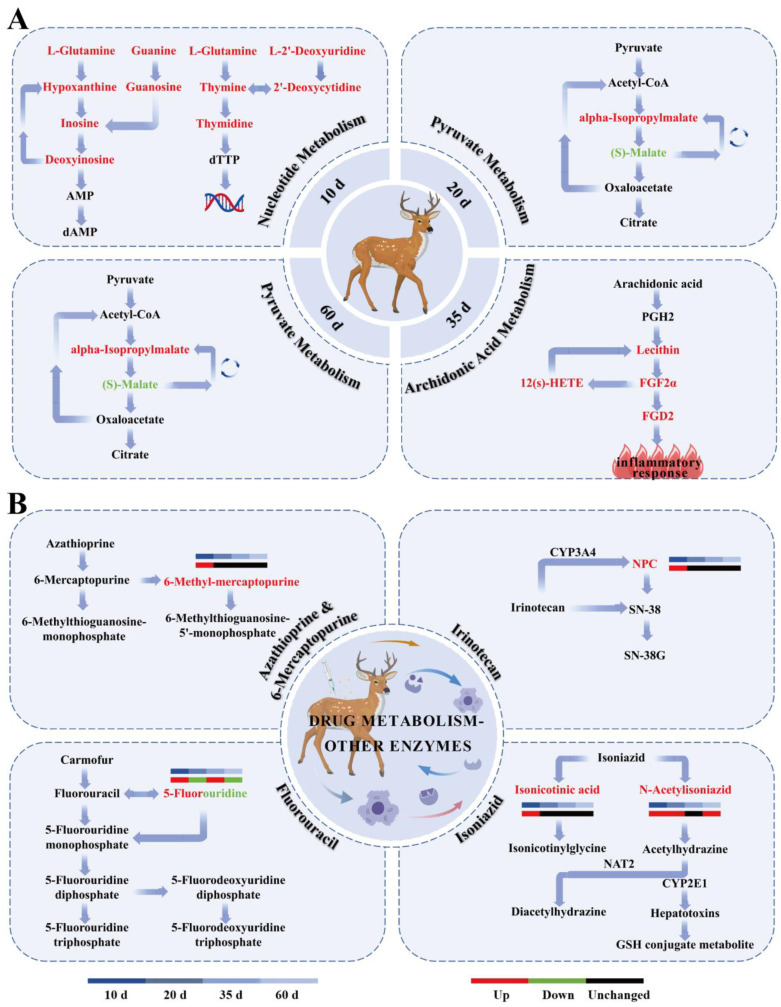
KEGG pathways of male sika deer at different time points and those involving common differential metabolites across time points. (**A**): KEGG pathways at different time points; (**B**): KEGG pathways involving common differential metabolites across time points. Red and green fonts indicate up-regulated and down-regulated pathways, respectively. Created with BioGDP.com [19].

KEGG pathway enrichment analysis was performed for differential metabolites identified at 10, 20, 35, and 60 days to explore their physiological relevance under exogenous progesterone treatment during the rutting period in male sika deer. At each time point, one pathway was significantly enriched (*p* < 0.05): nucleotide metabolism (ko01232; *p* = 0.027) at 10 days, pyruvate metabolism (ko00620; *p* = 0.022) at 20 days, arachidonic acid metabolism (ko00590; *p* = 0.025) at 35 days, and pyruvate metabolism (ko00620; *p* = 0.028) at 60 days (Figure 7 and Figure 8A). The drug metabolism–other enzymes pathway (ko00983) also showed enrichment across multiple time points. The most prominent changes and highest significance involved 5-fluorouridine and N-acetylisoniazid (Figure 8B). These findings suggest significant or trending effects on arachidonic acid metabolism, drug metabolism, and other enzyme pathways. This implies mild inflammatory responses and fluctuations in hepatic drug-metabolizing enzyme activity. Serum biochemical indicators were examined at 35 and 60 days post-administration to validate these metabolomics findings, confirm the absence of organ toxicity, and assess overall systemic safety.

### 3.4. Effects of Exogenous Progesterone on Serum Biochemical Indicators in Male Sika Deer

At 35 days post-treatment, the progesterone-treated group exhibited a significant decrease in the serum ALB/GLB ratio compared with the control group (*p* < 0.05). Although ALB levels decreased and GLB levels increased in the treatment group, these changes were not statistically significant (*p* > 0.05). By 60 days post-treatment, no significant inter-group differences were observed in any liver function indicators, including ALT, AST, ALB/GLB, and TBIL (*p* > 0.05). Similarly, no significant inter-group differences occurred in other biochemical indicators, including markers of kidney function, blood lipids, blood glucose, and electrolytes at either time point (*p* > 0.05) (Table 4; see Supplementary Table S1 for full details).

### 3.5. Effects of Exogenous Progesterone on Antler Performance in Male Sika Deer

As shown in Table 5, antler performance parameters did not differ significantly between the control and progesterone-treated groups (*p* > 0.05).

## 4. Discussion

This study examined the effects of a single subcutaneous injection of exogenous progesterone on rutting behavior, serum hormones, metabolome, biochemical indicators, and antler performance in adult male sika deer. The results demonstrate that exogenous progesterone effectively suppresses aggressive and mating behaviors during the rutting period, primarily through negative feedback regulation of the HPG axis, accompanied by coordinated endocrine and metabolic adjustments.

### 4.1. Effects of Exogenous Progesterone on Rutting Behavior in Male Sika Deer

Aggressive behavior scores in the progesterone-treated group were significantly reduced at 10, 20, 35, and 60 days post-injection (*p* < 0.05), while mating behavior scores showed a significant decline at 20, 35, and 60 days (*p* < 0.05). These findings provide clear evidence that exogenous progesterone effectively suppresses territoriality, sexual motivation, and aggression in male sika deer during the rutting period. Progesterone, though primarily associated with female reproduction, is also produced in males and exerts regulatory effects. Exogenous progesterone inhibits the HPG axis in males through negative feedback, reducing testosterone-mediated behaviors [21]. High progesterone concentrations may also disrupt androgen receptor signaling, reducing neuronal excitability in the hypothalamic-pituitary region and suppressing aggressiveness and sexual behavior [22,23]. The concurrent decline in aggressive and mating behaviors aligns closely with these mechanisms. The inhibitory effect of exogenous progesterone on male behavior is well-documented across species. For instance, progestin administration has been shown to effectively manage intraspecific aggression, roaming, and mounting in male dogs [24]. Similarly, in stallions, exogenous progesterone significantly suppresses circulating LH and testosterone levels, thereby mediating physiological and behavioral regulation [25]. Exogenous progesterone thus indirectly reduces energy expenditure and injury risk during the rutting period by suppressing aggressive and mating behaviors. This provides an effective endocrine approach for managing male aggression in farming. In contrast to permanent sterilization techniques commonly used in livestock such as swine and cattle [21], the present strategy aims for temporary behavioral modulation during a specific physiological stage, without inducing permanent loss of reproductive function. This distinction offers a distinct advantage for managing high-value animals such as sika deer.

A single progesterone injection provided stable hormonal exposure while avoiding repeated handling, restraint, or anesthesia, which are known to induce stress in large animals such as deer. Compared with traditional methods (e.g., feed restriction or physical isolation), progesterone treatment reduced monitoring and intervention needs during peak aggression. This approach lowered labor demands and operational costs and was not associated with observable adverse effects, such as emaciation or abnormal behaviors. Behavioral suppression persisted up to 60 days, likely due to residual effects after progesterone withdrawal. These may include delayed HPG axis resetting or neuroendocrine plasticity, resulting in sustained suppression of testosterone, LH, FSH, and PRL [7,26,27]. These findings indicate that progesterone intervention functions beyond short-term regulation. It may induce neuroendocrine reprogramming for long-term management in deer farms, especially high-density operations, to minimize fighting-related losses. Deeper mechanisms warrant further investigation to refine practical applications.

### 4.2. Effects of Exogenous Progesterone on Serum Hormone Levels in Male Sika Deer

To evaluate the regulatory effects of progesterone in male sika deer, we selected hormones representing multiple endocrine axes. These included core HPG axis hormones (GnRH, LH, FSH and T) to assess negative feedback inhibition; PRL because of its documented association to male behavior (e.g., aggressiveness) and metabolism; thyrotropin-releasing hormone (TRH) and T4 to examine thyroid axis cross-effects on energy metabolism and thermogenesis; growth hormone (GH) to detect interference with protein synthesis; estradiol (E2) for potential interconversions with T and progesterone; and dopamine (DA) for behavioral regulation. These choices reflected progesterone’s multi-target profile, extending beyond the reproductive axis through interactions with other axes via progesterone receptors (PR). The administration of exogenous progesterone exerted a profound suppressive effect on multiple hormonal axes, significantly reducing circulating levels of GnRH, LH, FSH, and T (*p* < 0.05). This suppression is attributable to the well-established negative feedback inhibition on the HPG axis, a mechanism explicitly confirmed in ruminants such as sheep [28,29]. Progesterone activates hypothalamic negative feedback via PR, inhibiting pulsatile GnRH release. This reduces pituitary LH and FSH secretion, decreasing testosterone synthesis in testicular Leydig cells. GnRH decreased significantly only at 10 and 20 days, likely due to the strongest initial inhibition, with gradual recovery as exposure waned. Progesterone levels were elevated at 10 and 20 days but normalized by 35 days, suggesting cessation of release around this time. Furthermore, the observed hormonal trajectory aligns with characteristic pharmacokinetic patterns of exogenous progesterone, which is rapidly cleared from circulation after treatment ends. For example, a pharmacokinetic study in mares reported that serum progesterone concentrations declined rapidly following implant removal [30]. Consequently, the return of progesterone to baseline by day 35 observed here can be explained by its rapid elimination kinetics.

PRL decreased significantly, indicating a complex regulatory mechanism. TRH normally stimulates PRL and influences male behavior and metabolism. In this study, however, the dissociation between elevated TRH and suppressed PRL suggests that exogenous progesterone overrides classical TRH-mediated regulation. This effect may occur through direct inhibition of pituitary PRL release via PR or through indirect pathways, such as enhanced sensitivity to dopaminergic inhibition [31]. This contradicts the classical TRH–PRL positive correlation, possibly because progesterone overrides TRH or because it is species-specific [32]. The inverse relationship between PRL and dopamine, whereby DA suppresses PRL secretion, appeared preserved, as DA levels remained unchanged, supporting a PR-driven mechanism of PRL suppression [33]. Although elevated PRL is known to inhibit GnRH secretion, the observed reduction in PRL did not lead to increased GnRH levels, likely because progesterone-mediated inhibition of the HPG axis remained dominant [29,34]. Together, these findings highlight progesterone-induced suppression of PRL through multi-axis endocrine crosstalk and support its potential role in regulating male behavior, although the extent to which these effects generalize across species warrants further investigation. This finding is in direct agreement with the work of Jeyaraj et al., who reported that progesterone significantly decreased serum levels of PRL, LH, and testosterone in male rats [35].

Progesterone may affect metabolism and tissue repair through interactions with the growth axis. In the present study, GH concentrations remained stable at 10 and 20 days post-treatment. This result is consistent with findings by Jeyaraj et al. in male rats, where exogenous progesterone did not significantly alter serum GH levels during the initial exposure period [35]. A notable finding of our study, however, was the dynamic change observed over a longer duration: GH levels declined significantly at day 35, followed by a marked elevation by day 60. These patterns likely reflect indirect effects, such as compensation for sustained testosterone decline (GH up-regulation maintains protein synthesis) or T4 decline (stimulating GH for energy regulation). These reflect post-release residual effects rather than direct inhibition during exposure. In ruminants, low testosterone and T4 from hormonal manipulation or stress often trigger compensatory GH elevation for protein synthesis and homeostasis [36]. T4 decline in heat stress similarly modulates GH, aiding energy adaptation [37]. This study offers new evidence of long-term multi-axis progesterone effects in male sika deer.

Elevated TRH and decreased T4 suggest interference with the thyroid axis, possibly via inhibited hepatic T4 conversion or PR-mediated hypothalamic TRH effects, weakening T4 negative feedback [38]. Unchanged estradiol (E2) and dopamine (DA) indicate regulatory selectivity. Despite a decline in testosterone, E2 did not decrease correspondingly, suggesting compensation through aromatization or weak progesterone effects on estrogen synthesis. DA, a PRL inhibitor linked to rutting motivation and aggression [39,40,41], remained stable. Thus, progesterone regulates PRL via DA-independent mechanisms. Behavioral suppression likely stems mainly from HPG-axis feedback and direct PRL effects, rather than from central dopaminergic changes. Behaviors and hormones (e.g., LH, FSH, T, PRL) remained lower than in controls for up to 60 days, likely due to residual effects such as receptor desensitization [42]. These interactions demonstrate broad progesterone effects in male sika deer, resembling sheep but with greater deer sensitivity [43]. This selectivity supports optimized intervention strategies, though transcriptomic integration is required for molecular insights.

### 4.3. Effects of Exogenous Progesterone on the Serum Metabolome in Male Sika Deer

Exogenous progesterone administration as a sustained-release injection significantly altered the serum metabolome of male sika deer during the rutting period. This was evidenced by differential metabolite accumulation across multiple time points and activation of key metabolic pathways. The observed metabolomic shifts extended beyond regulation of reproductive behavior, exerting systemic effects on energy metabolism and inflammatory signaling. These findings support progesterone’s role in maintaining physiological homeostasis via metabolic reprogramming and establish an interactive network with hormonal changes.

Multivariate analyses, including principal component analysis (PCA), partial least squares discriminant analysis (PLS-DA), and orthogonal PLS-DA (OPLS-DA), consistently demonstrated clear separation between the treatment and control groups. These analyses identified 1730 differential metabolites, predominantly lipids and lipid-like molecules (34.71%). This distribution aligns with progesterone’s steroid nature and is likely due to the regulation of hepatic lipid-synthesizing enzymes [44]. KEGG pathway enrichment analysis revealed significant activation of nucleotide metabolism at 10 d, accompanied by up-regulation of metabolites such as L-glutamine, thymidine, and deoxyinosine. This may stem from progesterone-induced inhibition of DNA synthesis, reshaping hepatic nucleotide networks and reallocating energy from high-energy synthesis to basal metabolism [45,46]. Pyruvate metabolism was altered at 20 and 60 d, with up-regulation of α-isopropylmalic acid and down-regulation of L-malic acid. These changes suggest that progesterone diverts glucose to the pentose phosphate pathway, increasing NADPH production for antioxidant defense [47,48]. Arachidonic acid metabolism was enriched at 35 d, with up-regulated derivatives including prostaglandin D2 and 12-HETE. This indicates that progesterone regulates inflammation via the cyclooxygenase pathway, mitigating rutting-period stress [49]. Common differential metabolites (e.g., combretastatin A4 and andrographolide) were enriched in the drug metabolism–other enzymes pathway. This reflects adaptive enzyme induction during progesterone exposure, likely involving hepatic cytochrome P450 activation to metabolize exogenous hormones [50]. These mechanisms arise from progesterone receptor (PR) expression in the liver and peripheral tissues, which mediates transcriptional regulation of amino acid and lipid catabolism. This reduces energy demands for behavior and maintains redox balance [51].

These metabolic changes are interconnected with significant reductions in HPG axis hormones (e.g., GnRH, LH, FSH, and T) via a hormone–metabolism interaction network [52]. Progesterone lowers testosterone-mediated energy demands through negative feedback on the HPG axis, redirecting carbon metabolism. For example, the early up-regulation of nucleotide metabolism at 10 d may reflect combined declines in GnRH and testosterone, leading to inhibition of DNA synthesis and facilitating metabolic adaptation to behavioral suppression [53,54]. Pyruvate metabolism alterations at 20 and 60 d reflect progesterone-driven diversion of glycolysis to the pentose phosphate pathway. These changes are associated with reductions in testosterone and PRL, the latter of which normally promotes hepatic gluconeogenesis [55,56]. Activation of arachidonic acid metabolism at 35 d may be linked to elevated TRH and reduced T4 concentrations, thereby stimulating lipid oxidation and inflammatory signaling as part of endocrine adaptation to sustained progesterone exposure [57,58].

Analysis of the 37 common differential metabolites detected across all time points revealed three principal abundance patterns: persistent up-regulation, persistent down-regulation, and stage-specific fluctuations. Persistently up-regulated metabolites (e.g., trans-chalcone, cortisone acetate, and andrographolide) remained elevated across all time points, indicating stable metabolic remodeling amid early progesterone increase and sustained reductions in LH, FSH, testosterone, and PRL. In contrast, persistently down-regulated metabolites were relatively few, exemplified by Arg–Tyr–Tyr, suggesting sustained inhibition of specific metabolic pathways under progesterone exposure. Most metabolites (e.g., combretastatin A4, tricarballylic acid, 5-fluorouridine, and spironolactone) showed alternating up- and down-regulation. Their change patterns aligned with pyruvate metabolism enrichment (20 and 60 d), arachidonic acid activation (35 d), GH fluctuations, and TRH/T4 changes, indicating phase-specific metabolic adjustments tied to hormonal shifts. Overall, these common metabolites comprise both stable (i.e., consistent differences from controls) and stage-specific variants. They reflect primary adaptation patterns associated with multi-axis hormonal changes induced by exogenous progesterone and provide a basis for elucidating its physiological roles in male sika deer.

Several limitations should be acknowledged, including the relatively small sample size (*n* = 6 per group) and reliance on the bovine KEGG database, which lacks sika deer–specific pathway annotations. These constraints warrant validation in larger cohorts with deer genome-assisted analysis. These findings align with those of Zhao et al. in male sika deer, in which progesterone down-regulated lipid-synthetic enzymes, consistent with the high proportion of lipid-differential metabolites (34.71%) observed here [59]. In contrast to rodent studies (e.g., Bales & Saltzman [60]), this work integrates multi-time-point metabolomics with HPG axis dynamics, uncovering temporal patterns in differential metabolites and advancing understanding of reproductive metabolic reprogramming in cervids. Such differences may arise from cervid seasonal specificity and the long-acting injection used here [61].

### 4.4. Effects of Exogenous Progesterone on Serum Biochemical Indicators in Male Sika Deer

At 35 days post-intervention, the serum ALB/GLB ratio was significantly reduced in the treatment group (*p* < 0.05). Although ALB tended to decrease and GLB to increase (*p* > 0.05), this ratio change suggests a mild inflammatory response or a transient adjustment in hepatic protein synthesis [62,63,64]. This finding aligns with the observed up-regulation of pyruvate metabolism in metabolomics data. This pathway is frequently activated during inflammation to support immune responses and oxidative stress management [65,66]. Up-regulation of 5-fluorouridine in the drug metabolism–other enzymes pathway suggests enhanced activity of hepatic drug-metabolizing enzymes, particularly cytochrome P450 isozymes [67,68]. Despite these metabolic adjustments, liver function enzymes, including ALT and AST, remained within normal ranges, indicating that the long-acting progesterone treatment induced only low-grade and reversible metabolic stress without evidence of overt hepatocellular injury. By 60 days post-administration, all serum biochemical indicators had normalized, showing no evidence of systemic toxicity.

Metabolomics data correspondingly revealed activation of arachidonic acid metabolism. This likely reflects hormone-mediated adaptive regulation of immune homeostasis rather than sustained pathological inflammation [49,69]. Within the drug metabolism–other enzymes pathway, fluctuations in metabolites such as 5-fluorouridine and N-acetylisoniazid have been associated with hepatotoxic responses in experimental models exposed to exogenous compounds. However, in the present study, no significant abnormalities were detected in ALT or AST levels, and the transient reduction in the ALB/GLB ratio observed at 35 days had fully recovered by 60 days. These observations indicate that progesterone-induced metabolic changes were mild, time-dependent, and reversible, without cumulative hepatotoxicity [70,71]. In marked contrast to the present findings, sustained progesterone or steroid hormone intervention is well-documented to induce hepatic burden or pathological injury in primates and rodents. For example, long-term androgen administration in rhesus monkeys led to a sustained decrease in HDL-C and significant dysregulation of lipoprotein metabolism [72]. Similarly, in rodent models, continuous treatment with steroid drugs or progesterone has been shown to cause marked elevations in serum ALT and increased inflammatory markers, indicating substantial hepatocellular injury [44,73].

Serum biochemical assessments provided independent validation of key metabolomic observations. Specifically, the reduction in the ALB/GLB ratio at 35 days coincided with activation of arachidonic acid metabolism, while normalization at 60 days was consistent with adaptive regulation of pyruvate metabolism. Additionally, no significant changes occurred in other indicators (myocardial enzymes, kidney function, blood lipids, electrolytes, or glucose) at either time point. Based on combined metabolomic and biochemical evidence, this study demonstrates a favorable short-term safety profile of exogenous progesterone administration in male sika deer. Nevertheless, given the multi-year production cycles typical of sika deer farming, the possibility of cumulative effects from repeated dosing remains and warrants further investigation through long-term, multi-cycle studies.

### 4.5. Effects of Exogenous Progesterone on Antler Performance in Male Sika Deer

Exogenous progesterone administration significantly reduced T, LH, and FSH levels. This prompted speculation that it might transiently disrupt antler regeneration cycles, analogous to its delay of estrus in females through HPG axis inhibition. However, no significant differences were observed between groups in antler yield, casting dates, or harvesting dates. These findings indicate that short-term (~35 days) progesterone exposure did not disrupt testosterone-dominated antler regeneration or adversely affect antler performance. This outcome is critical for sika deer farming, as velvet antler constitutes the primary economic product and yield stability directly influences revenue. Combined with the observed hormone–metabolism interactions and biochemical analyses, these results establish robust evidence for both safety and efficacy. Exogenous progesterone markedly suppresses endogenous androgens without inducing systemic metabolic disorders, hepatotoxicity, or reduced antler performance, confirming high safety and practical value at the tested dose and timing.

## 5. Conclusions

This study demonstrates that a single subcutaneous injection of exogenous progesterone (330 mg) effectively suppressed aggressive and mating behaviors in male sika deer during the rutting period, with effects lasting up to 60 days post-administration. This provides a practical management tool for high-density farming. The primary mechanism entailed negative feedback on the HPG axis, lowering GnRH, LH, FSH, PRL, and T levels. In addition to HPG axis modulation, progesterone interacted with other endocrine axes, as evidenced by increased TRH and decreased T4 concentrations within the thyroid axis, as well as time-dependent fluctuations in growth hormone levels. Within this endocrine framework, Non-targeted metabolomics revealed that progesterone-induced hormonal fluctuations triggered serum metabolic reprogramming. Differential metabolites were primarily enriched in nucleotide, pyruvate, and arachidonic acid metabolism pathways, reflecting underlying adaptations in energy reallocation and inflammation regulation. Although hormonal and metabolic changes were most pronounced early after intervention, biochemical indicators at 35 and 60 days—including liver and kidney function, myocardial enzymes, and blood lipids—remained normal, as did antler performance. This indicates no detectable damage to liver or kidney function and no impact on subsequent antler production under the tested regimen, indicating safety. In conclusion, by integrating endocrine, metabolic, and clinical safety data, this work elucidates the physiological basis of progesterone-mediated rutting suppression and confirms its dual benefit in safeguarding welfare and maintaining productivity, offering an economical and practical management strategy for the deer farming industry.

## Figures and Tables

**Table 1:** Rutting Behavior Scoring Criteria.

Score	Aggressive Behavior Description	Mating Behavior Description
0	No aggressive behavior	No behaviors related to rutting
1	Mild aggressive intent, such as lowering the head in threat	Occasional sniffing of the surroundings or low vocalization, without persistent behavior
2	Occasional aggressive behavior	Penile throbbing, nasal vocalization, tongue extension with vocalization, occasional rutting behavior
3	Relatively frequent aggressive behavior, with minor injury	Frequent walking, persistent vocalization or ground sniffing, with obvious rutting manifestations
4	Frequent and relatively intense aggressive behavior	Frequent stomping, persistent vocalization, etc., with diversified rutting behaviors
5	Persistent and intense aggressive behavior	Almost continuous strong rutting state, with long duration and high frequency of behaviors

The behavioral descriptions and scoring criteria of this standard were established based on the typical rutting behaviors of male sika deer (e.g., roaring, chasing, antler fighting), informed by preliminary observations of the study population.

**Table 2:** Number of significantly altered metabolites between the two groups of male sika deer at different time points.

Statistical Comparisons								
Statistically Significant Biochemicals	ANOVA Contrasts							
	Control/Treatment							
	10 d		20 d		35 d		60 d	
Total biochemicals ≤ 0.05	1321		444		403		469	
Biochemicals (↑↓)	858 ↑	463 ↓	269 ↑	175 ↓	182 ↑	221 ↓	310 ↑	159 ↓

↑ indicates up-regulation in the treatment group relative to the control group; ↓ indicates down-regulation.

**Table 3:** Common differential metabolites between the two groups of male sika deer across different time points.

Compound_ID	Name	log_2_FC			
		10 d	20 d	35 d	60 d
Com_1016_pos	Combretastatin A4	−3.53	1.48	−2.89	1.35
Com_908_pos	3-O-Methylbatatasin III	−3.86	1.15	−2.99	1.27
Com_544_pos	trans-Chalcone	1.47	1.87	1.36	1.33
Com_1561_pos	Cortisone acetate	5.18	3.04	1.40	3.63
Com_753_pos	gamma-Glutamyl-methionine	1.51	1.95	1.48	3.07
Com_3920_pos	4-Hydroxyoctanedioylcarnitine	−1.02	−3.03	−2.69	1.04
Com_3444_pos	Alfalone	3.75	1.79	1.69	1.00
Com_3310_pos	Alpinetin Methyl Ether	2.95	1.34	2.34	1.01
Com_1212_pos	(R)-Isomucronulatol	−2.17	−1.42	1.36	−1.39
Com_3652_pos	Bangangxanthone B	−2.90	1.93	−1.36	−2.94
Com_4972_pos	Incaspitolide E	3.80	3.28	1.56	3.66
Com_5143_pos	Arg Tyr Tyr	−1.27	−2.80	−1.01	−2.03
Com_5058_pos	Tyr Trp Thr	1.97	3.20	1.78	3.91
Com_5060_pos	CARDIVIN B	4.24	3.39	1.60	3.10
Com_4668_pos	Cacospongin D	2.21	−1.05	2.19	−2.02
Com_5157_pos	Xanthanthusin G	−2.42	1.71	1.54	1.37
Com_484_neg	4,5,7-Trimethoxyflavonol	−5.63	−1.15	1.02	−1.52
Com_294_neg	Sappanone A	1.39	−1.32	1.91	−1.15
Com_502_neg	Andrographolide	4.77	2.47	1.79	2.34
Com_89_neg	Tricarballylic acid	3.52	4.15	−2.58	6.68
Com_229_neg	5-Fluorouridine	5.20	−1.77	3.90	−1.72
Com_1609_neg	(ent-6alpha,7alpha)-6,7-Dihydroxy-16-kauren-19-oic acid	4.98	3.32	1.67	3.82
Com_2300_neg	1-Stearoylglycerophosphoserine	−2.13	−1.93	1.62	−2.92
Com_1907_neg	1-hexadecyl-glycero-3-phosphate	−2.04	−1.21	1.28	−1.55
Com_1875_neg	Glu-Gly-Trp	−2.39	1.45	−1.11	2.05
Com_2309_neg	11-Oxomogroside IA1	−2.06	−4.33	4.08	−2.03
Com_1566_neg	Dihydromelilotoside	2.22	2.38	−1.70	2.86
Com_1171_neg	Thalidomide	2.76	−2.85	1.17	−2.48
Com_1041_neg	4,7-Dihydro-5-(4-methyl-3-pentenyl)-1,2,3-trithiepin	2.87	1.26	−2.15	1.35
Com_936_neg	3-ethylphenyl Sulfate	3.55	1.14	1.09	1.80
Com_1666_neg	Hypoprotocetraric acid	1.65	1.25	1.72	1.72
Com_2009_neg	(3R)-1-Octen-3-yl-beta-primeveroside	−1.38	1.84	−1.17	3.73
Com_1987_neg	2-Phenylethyl beta-primeveroside	−1.28	1.80	−1.42	2.53
Com_2225_neg	Monnieraside II	−2.18	1.43	−3.24	1.34
Com_2078_neg	Lusitanicoside	−4.21	1.81	1.34	2.44
Com_1699_neg	11beta,13-dihydrolactucin 15-oxalate	1.85	1.86	−1.29	2.52
Com_1988_neg	Spironolactone	−2.00	1.37	−1.41	2.86

The greater the absolute value of log_2_FC, the more significant the fold change in the metabolite; the positive or negative sign indicates “up-regulation” or “down-regulation,” respectively.

**Table 4:** Comparison of serum biochemical indicators between the two groups of male sika deer at 35 d and 60 d.

Indicator		35 d		60 d	
		Control	Treatment	Control	Treatment
Liver function	ALB (g/L)	25.90 ± 1.07	25.00 ± 1.25	24.92 ± 0.88	23.53 ± 1.64
	GLB (g/L)	35.57 ± 1.23	37.58 ± 1.86	33.35 ± 1.14	32.35 ± 1.53
	ALB/GLB	0.72 ± 0.02	0.66 ± 0.01 *	0.75 ± 0.03	0.80 ± 0.10

* *p* < 0.05 compared with the control group at the same time point; the same below.

**Table 5:** Comparison of antler performance following exogenous progesterone application during the rutting period in male sika deer.

Group (*n* = 6)	Antler Casting Date	Antler Harvesting Date	Velvet Antler Weight (kg)
Control	4 May 2024–7 June 2024	26 June 2024–30 July 2024	1.772 ± 0.143
Treatment	1 May 2024–5 June 2024	22 June 2024–31 July 2024	1.789 ± 0.212
*p* value			0.41

## Data Availability

The datasets produced and/or analyzed during the current study are available from the corresponding author upon reasonable request.

## Supplementary Materials

The following supporting information can be downloaded at: https://www.mdpi.com/article/10.3390/ani16030488/s1, Table S1: Comparison of serum biochemical indicators between the two groups of male sika deer at 35 d and 60 d.
